# P32 Performance of a novel molecular test designed for point-of-care UTI diagnosis

**DOI:** 10.1093/jacamr/dlad077.036

**Published:** 2023-08-02

**Authors:** Ayako van der Goes, Joanna Diggle, Jeroen Nieuwland, Ali Roula, Emma Hayhurst

**Affiliations:** University of South Wales, UK; University of South Wales, UK; Public Health Wales, UK; University of South Wales, UK; Llusern Scientific, UK; University of South Wales, UK; Llusern Scientific, UK; University of South Wales, UK; Llusern Scientific, UK

## Abstract

**Objectives:**

To evaluate the performance of a novel LAMP-based molecular test for the diagnosis of UTIs. The Llusern Scientific UTI test system consists of a panel of six uropathogen tests, positive and negative controls and an analyser, ‘Lodestar DX’ (Figure 1). The analyser will read the reaction data and give the user a simple ‘positive’ or ‘negative’ read out for each uropathogen. The specific aim was to determine the sensitivity and specificity of the test system for the most common uropathogen, *Escherichia coli*.

**Figure 1.**
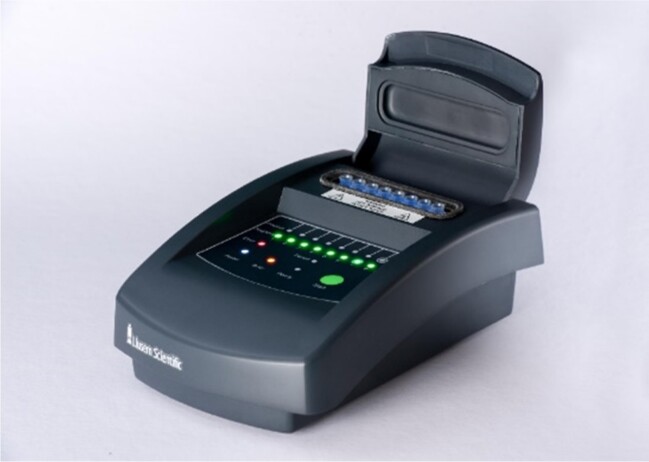
Lodestar DX.

**Methods:**

In total, 149 urine samples received by Public Health Wales laboratories, Cardiff were randomly selected; 1 μL of each urine sample was added directly to each pre-made *E. coli* LAMP test. *E. coli* LAMP tests were placed into the Lodestar DX analyser and run. Results were shown and recorded after 40 minutes as ‘positive’ or ‘negative’ according to the decision of Lodestar's algorithm. LAMP results were compared to standard culture results and sensitivity and specificity calculated.

**Results:**

Overall sensitivity of the *E. coli* LAMP test was 86.2%. Specificity was 88.3% (Table 1). Of the 14 samples positive in the LAMP test for *E. coli* but not recorded as *E. coli* using culture, 11 were from ‘mixed growth’ samples (Table 2).

**Table 1.** Sensitivity and specificity of *E. coli* LAMP test

	LAMP positive	LAMP negative	Total
Culture positive	25 (true positives)	4 (false negatives)	29
Culture negative	14 (false positives)	106 (true negatives)	120
Total	39	110	149
Overall sensitivity	((25/29) × 100) = 86.2%		
Overall specificity	((106/120) × 100) = 88.3%

**Table 2.** Results of LAMP compared with culture results

Culture result	*n*	*E. coli* Lodestar LAMP result		
		positive	inconclusive	negative
*E. coli* positive	29	25	0	4
Other pathogen positive	12	1	0	11
Mixed growth	61	11	0	50
No growth	47	2	0	45
Total	149	39	0	110

**Conclusions:**

The Llusern Scientific UTI test system shows great promise as a point-of-care diagnostic tool. Sensitivity and specificity for *E. coli* was high and the test system may be better at diagnosing mixed infections which represent a high proportion of all culture results. The test system is easy to use with no sample processing and minimal training requirements. Test results are available in 40 min, compared to 24–72 h for culture results. The performance of the test system for the detection of other uropathogens (*Klebsiella, Enterococcus*, *Pseudomonas, Proteus* and *S. saprophyticus*) is ongoing. Sensitivity can be further improved by refining the test method.

